# Simultaneous Liver–Kidney Transplantation from a Deceased Donor for Glycogen Storage Disease Type Ia: A Case Report

**DOI:** 10.70352/scrj.cr.26-0304

**Published:** 2026-08-26

**Authors:** Yuta Hirata, Yukihiro Sanada, Kiichiro Takadera, Ryosuke Akimoto, Takahiko Omameuda, Toshio Horiuchi, Noriki Okada, Taiichi Wakiya, Yasuharu Onishi, Yasunaru Sakuma, Hironori Yamaguchi

**Affiliations:** Department of Surgery, Division of Gastroenterological, General and Transplant Surgery, Jichi Medical University, Shimotsuke, Tochigi, Japan

**Keywords:** glycogen storage disease type Ia, simultaneous liver–kidney transplantation, liver transplantation, kidney transplantation

## Abstract

**INTRODUCTION:**

Glycogen storage disease type Ia (GSD Ia) is an indication for simultaneous liver–kidney transplantation (SLKT) due to multiple hepatocellular adenomas and chronic renal failure. In this report, we describe a deceased-donor SLKT in a patient with GSD Ia who presented with hypoglycemic attacks, progressive multiple hepatocellular adenomas, and chronic renal failure.

**CASE PRESENTATION:**

The patient was diagnosed with hepatomegaly at the age of 1 year and 6 months, with a diagnosis of GSD Ia, and was started on a special diet. She had been aware of liver tumors since 24 years of age. At 29 years of age, she underwent left lateral segmentectomy and radiofrequency ablation for multiple liver tumors. She began to suffer from renal dysfunction around 39 years of age, and then at 40 years of age, she was diagnosed with chronic renal failure and therefore was started on hemodialysis. She began having recurrent hypoglycemic attacks after the introduction of dialysis. Due to repeated hypoglycemic attacks, multiple hepatocellular adenomas, and chronic renal failure, she was deemed eligible for SLKT and thus was registered for deceased-donor SLKT at 41 years of age. She underwent SLKT at 46 years of age. The liver graft weight was 1060 g, and the graft-to-recipient body weight ratio was 2.41. The kidney graft weight was 170 g. The weight of the excised liver was 2710 g. Although no malignant findings were observed in the excised liver, multiple hepatocellular adenomas were identified. She was able to urinate independently immediately after the SLKT and was able to discontinue dialysis immediately afterward. Immunosuppressive therapy was initiated with basiliximab, ciclosporin A, methylprednisolone, and mycophenolate mofetil (MMF). However, due to abdominal pain and liver dysfunction caused by MMF, she was switched to mizoribine on POD 46, and her abdominal pain improved thereafter. She progressed well and was discharged on POD 49. Her liver and kidney functions remained good approximately 2 years after the SLKT.

**CONCLUSIONS:**

Adult patients with GSD Ia require transplantation due to hypoglycemic attacks, multiple liver tumors, and chronic renal failure.

## Abbreviations

ALT
: alanine aminotransferase
CMV
: cytomegalovirus
CsA
: ciclosporin A
EBV
: epstein-barr virus
GSD Ia
: glycogen storage disease type Ia
IVC
: inferior vena cava
KT
: kidney transplantation
LT
: liver transplantation
MMF
: mycophenolate mofetil
PV
: portal vein
SLKT
: simultaneous liver–kidney transplantation
Tac
: tacrolimus

## INTRODUCTION

Glycogen storage disease type I is a rare metabolic disorder (1 in 100000) with an autosomal recessive inheritance, in which an abnormal glucose metabolism results in the accumulation of glycogen in living tissues, thus causing a variety of symptoms. In addition, glycogen storage disease type I is associated with multiple hepatocellular adenomas and hepatocellular carcinoma.^1,2)^ GSD Ia is caused by a deficiency of glucose-6-phosphatase, which is involved in the final stage of glucose production from the hepatic glycogen stores.^3)^ GSD Ia presents with hepatomegaly, hypoglycemia, lactic acidosis, hyperlipidemia, hyperuricemia, renal disease, and hepatocellular adenoma.^2)^ GSD Ia is an indication for SLKT due to multiple hepatocellular adenomas and chronic renal failure. In this report, we describe a deceased-donor SLKT in a patient with GSD Ia who presented with hypoglycemic attacks, progressive multiple hepatocellular adenomas, and chronic renal failure.

## CASE PRESENTATION

**Fig. 1:**
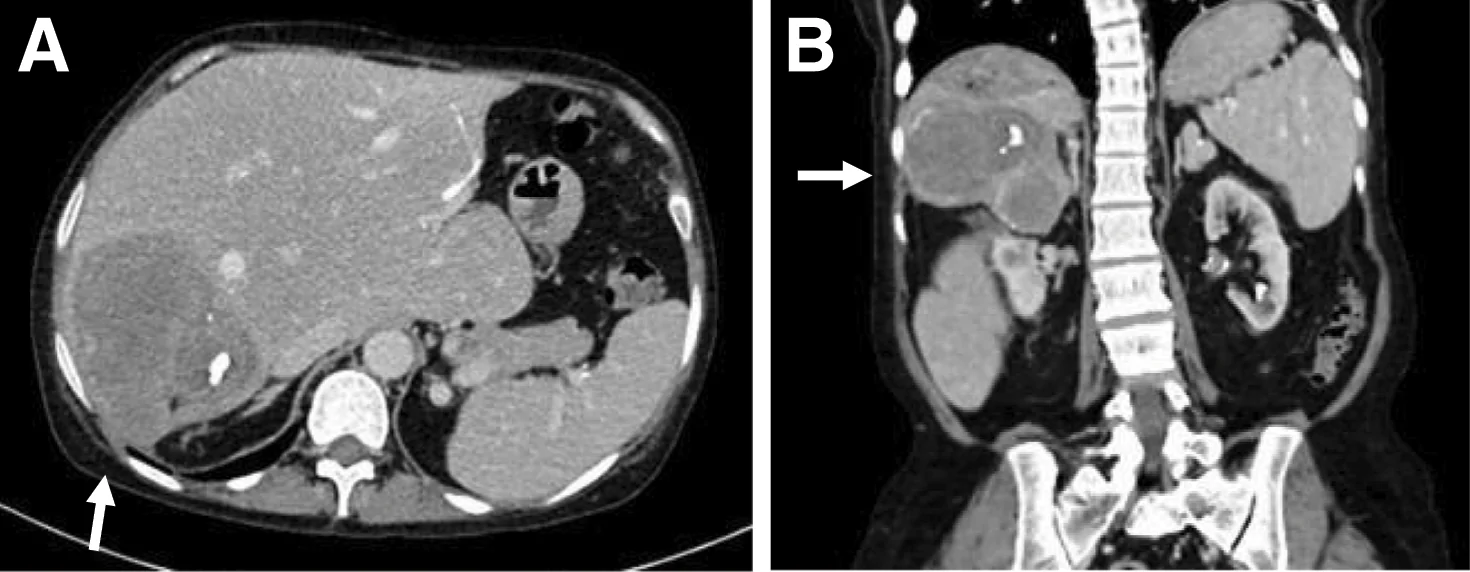
The CT findings of the tumor-like lesions containing calcifications and fat observed in the S7 region of the liver before the SLKT (**A** and **B**). SLKT, simultaneous liver-kidney transplantation

**Fig. 2:**
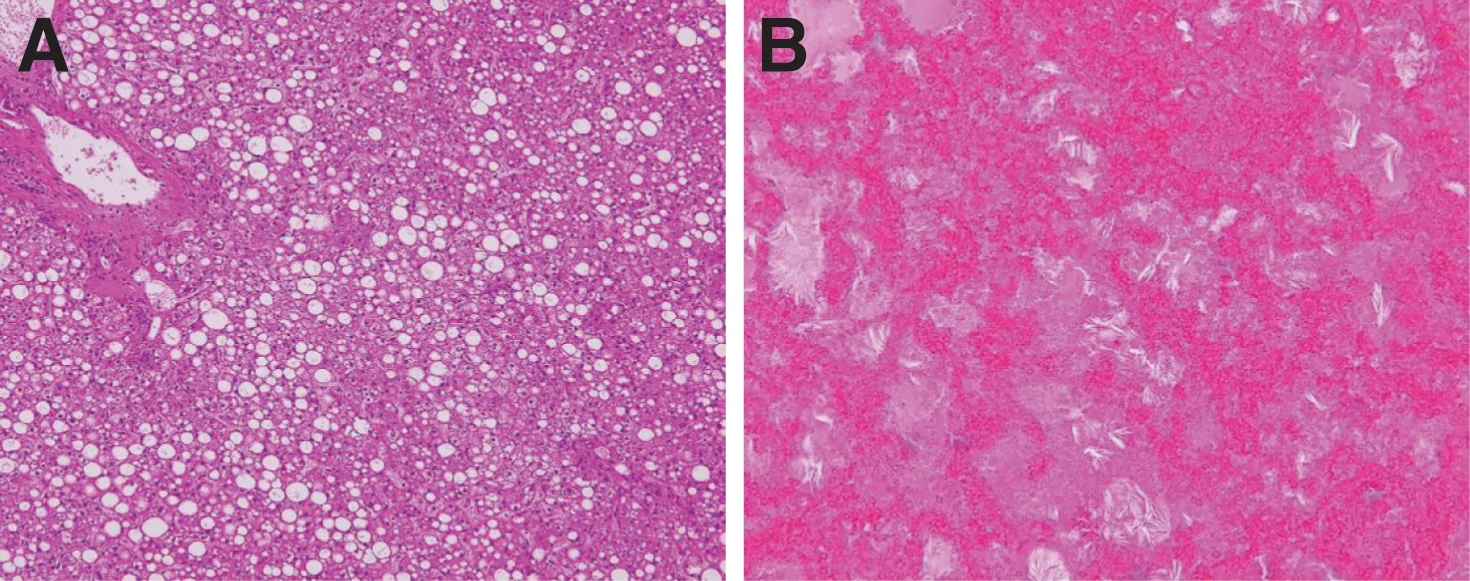
Hepatocytes have a pale, foamy, granular cytoplasm. The findings, which included predominantly macropatic steatosis, were consistent with those seen in GSD Ia (**A**). The hepatocellular adenoma showed signs of hyaline necrosis, with fibrosis noted in the surrounding area. These findings were consistent with hepatocellular adenoma (**B**). GSD Ia, glycogen storage disease type Ia

The patient was diagnosed with hepatomegaly at the age of 1 year and 6 months, with a diagnosis of GSD Ia, and was started on a special diet. She had been aware of liver tumors since 24 years of age. At 29 years of age, she underwent left lateral segmentectomy and radiofrequency ablation for multiple liver tumors. She began to suffer from renal dysfunction around 39 years of age, and then at 40 years of age, she was diagnosed with chronic renal failure and therefore was started on hemodialysis. She began having recurrent hypoglycemic attacks after the introduction of dialysis. Due to repeated hypoglycemic attacks, multiple hepatocellular adenomas, and chronic renal failure, she was deemed eligible for SLKT and thus was registered for deceased-donor SLKT at 41 years of age (**Fig. 1**). She underwent SLKT at 46 years of age (**Table 1**). The liver graft weight was 1060 g, and the graft-to-recipient body weight ratio was 2.41. The kidney graft weight was 170 g. The weight of the excised liver was 2710 g. Although no malignant findings were observed in the excised liver, multiple hepatocellular adenomas were identified (**Fig. 2**). For the recipient operation, an inverted T incision was made. Total hepatectomy was performed. Hepatic vein reconstruction was performed between the recipient’s venoplasty of the left, middle, and right hepatic veins and the graft’s suprahepatic IVC. The PV reconstruction was performed between the recipient’s main PV and the graft’s main PV. Hepatic artery reconstruction was performed between the recipient’s branch patch of the common hepatic artery and the splenic artery and the graft’s right hepatic artery. Biliary reconstruction was performed with a choledochocholedochostomy. The right iliac fossa kidney transplant was successfully performed. The operative time for LT was 8 hours 56 minutes and for KT 2 hours 56 minutes.

**Table 1:** Demographic data of liver function tests and metabolic parameters

	At the time of deceased-donor SLKT of registration	At the time of SLKT
Total bilirubin (mg/dL)	0.2	0.4
Aspartate aminotransferase (U/L)	37	27
Alanine aminotransferase (U/L)	24	19
Albumin (g/dL)	4.2	4.8
Prothrombin time-international normalized ratio	0.98	1.04
Glucose (mg/dL)	79	44
Lactate (mmol/L)	–	5.8
Triglycerides (mg/dL)	641	323
Uric acid (mg/dL)	7.7	1.6
Creatine (mg/dL)	6.73	1.96

SLKT, simultaneous liver–kidney transplantation

**Fig. 3:**
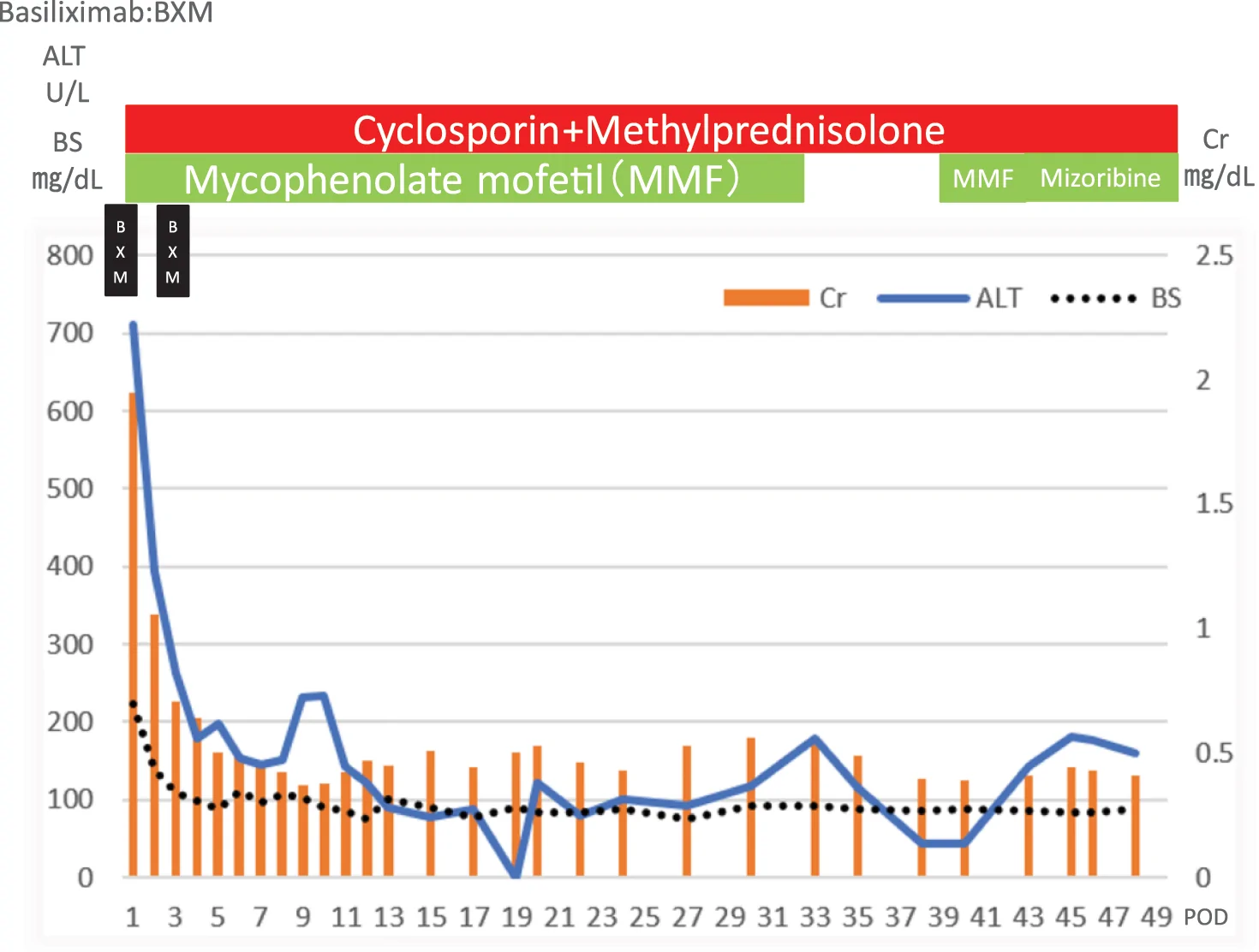
The postoperative course of the SLKT. SLKT, simultaneous liver-kidney transplantation

**Fig. 4:**
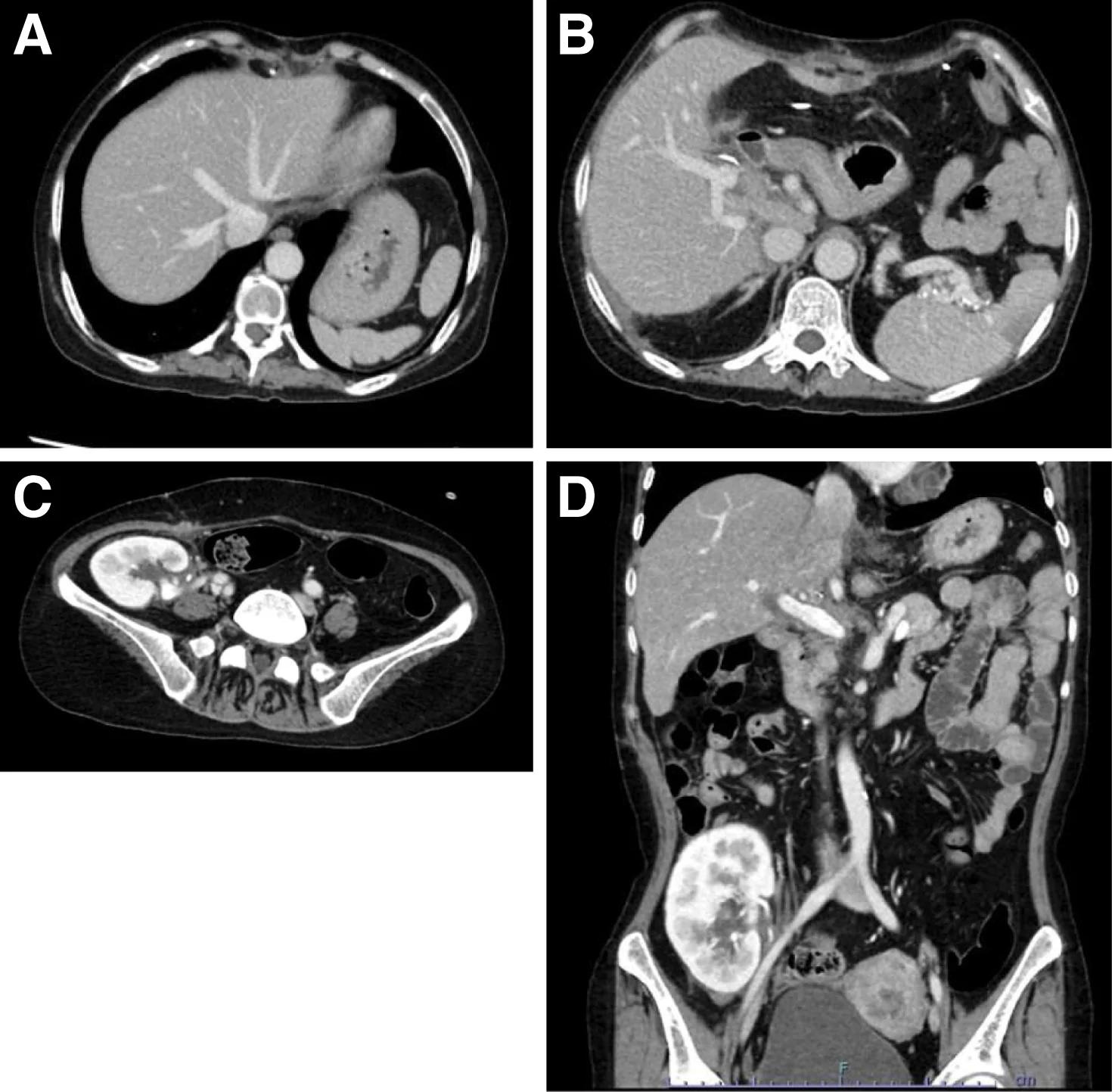
The CT findings of the liver and kidney after the SLKT (**A**, **B**, **C** and **D**). SLKT, simultaneous liver-kidney transplantation

She was able to urinate independently immediately after the SLKT and was able to discontinue dialysis immediately afterward. Immunosuppressive therapy was initiated with basiliximab, CsA, methylprednisolone, and MMF. On POD 33, a contrast-enhanced abdominal CT scan revealed no findings suggestive of biliary complications. Blood test results on POD 40 showed no evidence of either CMV or EBV infection. On POD 43, the ALT level increased to 143 U/L. Due to abdominal pain and liver dysfunction caused by MMF, she was switched to mizoribine on POD 46, and her abdominal pain improved thereafter. She progressed well and was discharged on POD 49 (**Fig. 3**). On POD 80, the ALT level improved to 30 U/L. Her liver and kidney functions remained good approximately 2 years after the SLKT (**Fig. 4**).

## DISCUSSION

The indications for LT in GSD I include growth retardation, hepatocellular adenoma, and hepatocellular carcinoma, while those in GSD III or GSD IV include cirrhosis and liver failure. The indications for LT in GSD Ia include uncontrolled hypoglycemic episodes and multiple hepatocellular adenomas. Takahashi et al. reported that hemodialysis stimulates the glycolytic pathway within red blood cells, thus leading to hypoglycemia as glucose is taken up and consumed by red blood cells.^4)^ It is believed that the initiation of hemodialysis exacerbates hypoglycemic episodes through the mechanisms described above. The long-term outcomes of LT for GSD Ia are favorable.^5,6)^ According to a report by the Japanese Liver Transplantation Society, deceased-donor LT for GSD has been performed in 5 cases. Additionally, the indication for KT in patients with GSD is end-stage renal failure.^7)^ We believe that SLKT should be considered when a patient with GSD Ia develops end-stage renal failure, uncontrolled hypoglycemic episodes, and multiple hepatocellular adenomas. There have been 54 cases of deceased-donor SLKT reported in Japan. The 5-year survival rate for SLKT in Japan is favorable, at 85.2%. Reports of deceased-donor SLKT for GSD have been sporadically observed overseas, with improvements in both the liver and kidney function reported in SLKT patients^7–11)^ (**Table 2**). When performing organ transplantation in patients with hepatic and renal failure, the question arises as to whether to perform an LT first, followed by a KT while the patient is on the waiting list, or to perform an SLKT. Although there is no significant difference in the 5-year survival rates between the 2 approaches, it has been reported that chronic rejection of the transplanted kidney and chronic renal dysfunction are milder in patients who undergo SLKT and that the loss of the kidney function is also less common.^12)^ This case was determined to be a candidate for deceased-donor SLKT due to recurrent hypoglycemic episodes following the initiation of dialysis for chronic renal failure and multiple hepatocellular adenomas present since the patient’s twenties. As there is no established immunosuppressive therapy protocol for SLKT, this case followed the immunosuppressive therapy protocol used for KT. GSD and Tac administration have been reported to increase the risk of acute pancreatitis.^13,14)^ There have been reports of SLKT in patients with GSD Ia in whom CsA was administered instead of Tac, and no complications of acute pancreatitis were observed.^7,10)^ This case presented with hypertriglyceridemia, a risk factor for acute pancreatitis. Considering the risk of acute pancreatitis, the immunosuppressive therapy protocol for this case was changed from Tac to CsA. Following SLKT, the patient did not develop acute pancreatitis for 2 years.

**Table 2:** Previous reports of SLKT patients for GSD

Case	Authors/Years	GSD Type	Age at diagnosis (years)	Age at the time of transplantation (years)/Sex	Indications for transplantation	Immunosuppressive therapy	Postoperative complication	Outcome
1	Matern D/ 1999^8)^	Ia	–	26/Male	Poor metabolic control Hepatocellular adenoma Chronic renal failure	–	Chronic rejection	Survival 11POY
2	Lee PJ/ 2004^9)^	Ia	9	33/Female	Poor metabolic control Chronic renal failure	Tac PSL	None	Survival 1POY
3	Panaro F/ 2004^7)^	Ia	–	25/Female	Hepatocellular adenoma Chronic renal failure	CsA PSL	Lactic acidosis during SLKT	Survival 4POM
4	Belingheri M/ 2007^10)^	Ia	1	19/Female	Poor metabolic control Chronic renal failure	CsA PSL MMF	None	Survival 4POY
5	Marega A/2011^11)^	Ia	2	30/Female	Hepatocellular adenoma Chronic renal failure	Tac PSL MMF	None	Survival 6POM

CsA, ciclosporin A; GSD, glycogen storage disease; MMF, mycophenolate mofetil; POY, post-operative year; POM, post-operative month; PSL, prednisolone; SLKT, simultaneous liver- kidney transplantation; Tac, tacrolimus

## CONCLUSIONS

Adult patients with GSD Ia require transplantation due to hypoglycemic attacks, multiple liver tumors, and chronic renal failure.

## DECLARATIONS

### Funding

We declare that we have no funding.

### Authors’ contributions

Study design: Y. H. and H. Y.

Data collection: Y. H., Y. Sanada., K. T., R. A., T. O., T. H., N. O., T. W., Y. O., and Y. Sakuma.

Data analysis: Y. H. and H. Y.

Data interpretation: Y. H. and H. Y.

Preparation of manuscript: Y. H.

Literature analysis: Y. H.

### Availability of date and materials

We confirm that the data supporting the findings of this study are available within the article and its supplementary materials.

### Ethics approval and consent to participate

All procedures performed in studies involving human participants were in accordance with the ethical standards of the institutional research committee and with the 1964 Declaration of Helsinki and its later amendments or comparable ethical standards. Informed consent was obtained from the participant included in the study.

### Consent for publication

Consent for publication was obtained from the participant included in the study.

### Competing interests

We declare that we have no competing interests.

## References

[1] Rake JP, Visser G, Labrune P, et al. Glycogen storage disease type I: diagnosis, management, clinical course and outcome. Results of the European study on glycogen storage disease type I (ESGSD I). Eur J Pediatr 2002; 161:S20–34. 12373567 10.1007/s00431-002-0999-4

[2] Franco LM, Krishnamurthy V, Bali D, et al. Hepatocellular carcinoma in glycogen storage disease type Ia: a case series. J Inherit Metab Dis 2005; 28:153–62. 15877204 10.1007/s10545-005-7500-2

[3] Marcolongo P, Fulceri R, Gamberucci A, et al. Multiple roles of glucose-6-phosphatases in pathophysiology: state of the art and future trends. Biochim Biophys Acta, Gen Subj 2013; 1830:2608–18. 10.1016/j.bbagen.2012.12.013 23266497

[4] Takahashi A, Kubota T, Shibahara N, et al. The mechanism of hypoglycemia caused by hemodialysis. Clin Nephrol 2004; 62:362–8. 15571181 10.5414/cnp62362

[5] Labrune P. Glycogen storage disease type I: indications for liver and/or kidney transplantation. Eur J Pediatr 2002; 161:S53–5. 12373572 10.1007/s00431-002-1004-y

[6] Davis MK, Weinstein DA. Liver transplantation in children with glycogen storage disease: controversies and evaluation of the risk/ benefit of this procedure. Pediatr Transplant 2008; 12:137–45. 18307661 10.1111/j.1399-3046.2007.00803.x

[7] Panaro F, Andorno E, Basile G, et al. Simultaneus liver-kidney transplantation for glycogen storage disease type Ia (von Gierke’s disease). Transplant Proc 2004; 36:1483–4. 15251364 10.1016/j.transproceed.2004.05.070

[8] Matern D, Starzl TE, Arnaout W, et al. Liver transplantation for glycogen storage disease types I, III, and IV. Eur J Pediatr 1999; 158:S43–8. 10603098 10.1007/pl00014320 PMC3006437

[9] Lee PJ, Muiesan P, Heaton N. Successful pregnancy after combined renal-hepatic transplantation in glycogen storage disease type Ia. J Inherit Metab Dis 2004; 27:537–8. 15334735 10.1023/b:boli.0000037397.39725.57

[10] Belingheri M, Ghio L, Sala A, et al. Combined liver-kidney transplantation in glycogen storage disease Ia: a case beyond the guidelines. Liver Transpl 2007; 13:762–4. 17457869 10.1002/lt.21147

[11] Marega A, Fregonese C, Tulissi P, et al. Preemptive liver-kidney transplantation in von Gierke disease: a case report. Transplant Proc 2011; 43:1196–7. 21620087 10.1016/j.transproceed.2011.03.003

[12] Eason JD, Gonwa TA, Davis CL, et al. Proceedings of consensus conference on simultaneous liver kidney transplantation (SLK). Am J Transplant 2008; 8:2243–51. 18808402 10.1111/j.1600-6143.2008.02416.x

[13] Ai J, He W, Huang X, et al. A case report of acute pancreatitis with glycogen storage disease type IA in an adult patient and review of the literature. Medicine (Baltimore) 2020; 99:e22644. 33080702 10.1097/MD.0000000000022644 PMC7571931

[14] Liu XH, Chen H, Tan RY, et al. Acute pancreatitis due to tacrolimus in kidney transplant and review of the literature. J Clin Pharm Ther 2021; 46: 230–5. 32949156 10.1111/jcpt.13269

